# Tissue-Specific Chromatin Accessibility Regions and Transcription Factor Binding Sites in Pig Brain and Endocrine Tissues

**DOI:** 10.1007/s12035-025-05128-5

**Published:** 2025-06-07

**Authors:** Siriluck Ponsuksili, Frieder Hadlich, Nares Trakooljul, Shuaichen Li, Henry Reyer, Michael Oster, Klaus Wimmers

**Affiliations:** 1 Research Institute for Farm Animal Biology (FBN), Wilhelm-Stahl-Allee 2, 18196 Dummerstorf, Germany; 2 Faculty of Agricultural and Environmental Sciences, University Rostock, 18059 Rostock, Germany

**Keywords:** Porcine, ATAC-seq, HPA axis, Binding motifs, Endocrine tissues, Brain tissues

## Abstract

The cognitive and regulatory processes within higher-order brain structures that regulate the hypothalamic–pituitary–adrenal (HPA) axis and the limbic system orchestrate a complex stress response system. In order to address this, we collected 48 tissue samples from the amygdala (Amy), hippocampus (Hip), thalamus (Tal), hypothalamus (HT), pituitary gland (PG) and adrenal gland (AG). We applied ATAC-seq, a method for profiling accessible chromatin, to map the epigenetic landscape of these brain and endocrine tissues in pigs and generate foundational baseline chromatin accessibility datasets that can serve as a reference for future studies. A total of 321,584 consensus peaks, representing open chromatin regions across various samples and tissues in the pig genome, were identified. Screening for transcription factor binding motifs within these chromatin-accessible regions revealed 377 significantly enriched motifs in at least one tissue (*p* ≤ 0.001). Among the 93 motifs enriched in only one tissue, some showed concordant expression of their corresponding transcription factors, including *GRHL2* and *KLF5* in the PG, and *GATA4/6*, and *HAND2* in the AG. Differentially accessible regions (DARs), particularly in promoter regions, between brain and endocrine tissues were identified, with functional specificities in the AG, including cortisol synthesis and secretion, as well as tyrosine metabolism. The cytokine-cytokine receptor interaction and neuroactive ligand-receptor interaction pathways showed greater enrichment and open chromatin accessibility in brain regions compared to endocrine tissues (PG or AG). This study provides valuable insights into brain transcriptional regulation and adds a novel layer of information for future research on genetic improvement and animal welfare.

**Supplementary Information:**

The online version contains supplementary material available at 10.1007/s12035-025-05128-5.

## Introduction

The hypothalamic–pituitary–adrenal (HPA) axis is a complex neuroendocrine system responsible for maintaining physiological homeostasis [1]. It involves intricate interactions between the hypothalamus, pituitary gland and adrenal glands, regulating processes such as stress response, digestion, immune function and mood [2]. The amygdala and hippocampus, both anatomically adjacent limbic regions in the forebrain, play distinct roles in emotional processing. The amygdala is crucial for prioritizing emotionally significant information, including emotion and motivation [3, 4], whereas the hippocampus is essential for modulating emotional memories [5]. Together, these structures are integral to feedback regulation of the HPA axis, with the hippocampus exerting an inhibitory influence on the hypothalamic paraventricular nucleus (PVN) via trans-synaptic pathways [6]. Previous studies in pigs have linked coping behaviors to molecular changes, particularly in neuroinflammation, glutamate and GABA metabolism in the amygdala and hippocampus, as well as enrichment in glucocorticoid receptor signaling, energy metabolism and NGF signaling in the HPA axis [7, 8].

Our previous studies identified epigenetic profiles including DNA methylation and small non-coding RNA expression in key structures mediating adaptive responses in pigs, including the hippocampus, amygdala, hypothalamus and adrenal gland, as effectors of the limbic system and the HPA axis [9, 10]. Epigenetic modifications including DNA methylation are believed to play a crucial role in regulating gene expression by controlling chromatin accessibility and guiding the binding of sequence-specific transcription factors [11]. The Farm Animal Genotype-Tissue Expression (FarmGTEx) project reported genetic regulatory effects across pig tissues [12]. The influence of regulatory variants or elements on molecular phenotypes depends not only on the tissue type but also on developmental stages [13]. In particular, the accessibility of *cis*-regulatory elements (CREs) is closely linked to transcriptional activity [14] thereby acting as a mechanism for the spatiotemporal control of gene expression in various biological processes and cell types. Several assays have been developed to decipher the epigenetic landscape using the latest high-throughput sequencing technologies, including Assay of Transposase Accessible Chromatin sequencing (ATAC-seq) [15, 16]. ATAC-seq is a powerful method for the identification of open chromatin regions and *cis*-regulatory motifs irrespective of the chemical nature of the epigenetic modification [15, 16]. Using ATAC-seq, a map of chromatin accessibility in two cell types (neurons and non-neurons) across 14 distinct human brain regions was reported, thereby demonstrating the importance of cell-type-specific epigenomic profiling in understanding the complex regulatory mechanisms in the brain and their potential role in neurophysiology [17]. Chromatin accessibility, an important epigenetic mechanism of gene regulation, directly influences the recognition and binding of TFs, and, thus, it is crucial for gene expression. However, knowledge of dynamic changes in chromatin accessibility across different brain regions and endocrine tissues—particularly in pigs—remains limited. Recent ATAC-seq studies have primarily focused on pig muscle tissue [18–20], and liver and CD4⁺/CD8⁺ T cells [21]. One other study examined chromatin accessibility across muscle, liver and brain without specifying the anatomical subregions of the brain [22].

We applied ATAC-seq profiling of accessible chromatin to generate baseline chromatin accessibility datasets from the pig’s limbic system and HPA/stress axis organs—the amygdala (Amy), hippocampus (Hip), thalamus (Tal), hypothalamus (HT), pituitary gland (PG) and adrenal gland (AG), derived from the same eight female pigs. A systematic analysis of the dataset identified a total of 321,584 consensus peaks, with 51,056 showing tissue-specific patterns. We also predicted transcription factors (TFs) binding sites from the consensus peak regions specific to distinct tissues. This study provides a valuable resource for understanding brain transcriptional regulation and serves as a novel layer of information for future research on genetic improvement and animal welfare in pig.

## Materials and Methods

### Animal Care and Tissue Sampling

The study was carried out using tissues sampled from eight female German Landrace (GL) pigs, with an average age of 170 ± 14 days and an average weight of 105 ± 8 kg. The animals belonged to the herd book herd owned by the Research Institute of Farm Animal Biology (FBN) and were housed in the FBN’s Pig Experimental Facilities. The animals were stunned and slaughtered in the morning between 8:00 and 10:00 at the FBN experimental abattoir, where adrenal gland and brain tissues—including the Amy, Hip, HT, PG and Tal—were collected. Different brain regions were dissected with the aid of the pig brain atlas [23]. Animal care and tissue collection procedures were conducted in compliance with European Directive 2010/63/EU on the protection of animals used for scientific purposes, where applicable, and Council Regulation (EC) No 1099/2009 on the protection of animals at the time of killing. Animal handling and humane slaughter were performed following European legislation and ethical guidelines. All necessary measures were taken to minimize pain and discomfort, ensuring adherence to best practices in animal research as outlined in the ARRIVE 2.0 guidelines.

To isolate nuclei from tissue, the tissues were slowly frozen in a cryoprotective medium consisting of 10% DMSO in fetal bovine serum (FBS). The pieces of tissues were immersed in the freezing medium in cryovials. The cryovials were placed in an isopropanol-filled freezing container to achieve a controlled freezing rate of – 1 °C per minute, and transferred to a – 80 °C freezer, the samples were stored long-term in the – 80 °C freezer for further analyses.

### Nuclei Purification, Transposition, and Library Preparation

The nuclear fraction was purified and enriched by density gradient centrifugation using iodixanol solution to remove mitochondria. Nuclei were counted using Trypan blue exclusion to determine the optimal number of viable nuclei (> 50,000) required for transposition. Transposition was performed using the Illumina Tagment DNA Enzyme and Buffer Kit (Tn5 Transposase/Tagment DNA Enzyme 1) according to the manufacturer’s protocol (Illumina, San Diego, CA, USA). To enhance nuclear permeabilization and protocol efficiency, Tween 20 and digitonin were added to the transposition reaction. Transposed DNA fragments were immediately purified using the MiniElute PCR Purification Kit (QIAGEN, Germany) and further amplified by PCR using Illumina Unique Dual Indexes (UDIs). The resulting libraries were purified and sequenced using paired-end sequencing (2 × 71 bp reads) on an Illumina NextSeq 2000 at the FBN.

### ATAC-Seq Data Processing and Differential Chromatin Accessibility Analysis

For differential open chromatin analysis, a sequencing depth of 50 million paired-end reads per sample was targeted. ATAC-seq data were processed using the nf-core/atacseq pipeline from NEXTFLOW (version 2.1.2). Raw sequencing reads were trimmed to remove adapters and low-quality bases using Trim Galore (version 0.6.7), a wrapper around Cutadapt (version 3.4) and quality was assessed with FastQC (version 0.11.9). The trimmed reads were then aligned to the pig reference genome (Sscrofa11.1) using the Burrow-Wheeler Aligner (BWA, version 0.7.17-r1188) with default settings. Any PCR duplicates were marked with Picard’s MarkDuplicates tool (version 3.0.0) to produce filtered BAM files. Reads mapping to the mitochondrial genome were excluded from further analysis. Insert sizes were calculated, and BAM files were prepared using SAMtools (version 1.17). Peaks were identified using model-based analysis for ChIP-Seq 2 (MACS2, version 2.2.7.1), a widely used software tool that performs peak calling—a computational process used to detect regions of the genome with significant enrichment of sequencing reads, indicating open chromatin or regulatory activity. Peak calling was carried out using the –format BAMPE option with default settings, and duplicate reads were removed to minimize artifacts. To assess the quality of the sequencing libraries, the transcription start site (TSS) enrichment score was calculated, providing a measure of signal-to-noise ratio and overall chromatin accessibility profiling quality.

### Calculation of Consensus Binding Site Intervals

MACS2 narrow peaks are counted in binding site intervals using the dba.count function from the R package DiffBind (version 3.14.0). For this analysis, fixed-width regions were defined by extending 75 base pairs on either side of the peak summits—the points of highest signal enrichment within each peak. Only peaks with a minimum read count of 10 and present in at least two overlapping peaksets were included. Furthermore, regions not being represented by 75% of samples in any group or falling below the threshold value of 100 reads were disregarded. Heatmap profiles were created using deeptools (version 3.5.1) and R package ggplot2. All annotations relied on ensEMBL version 112.

### Identification of Tissue-Specific Chromatin Accessible Regions

Tissue-specific chromatin accessible regions were identified using a Shannon entropy-based method [24], which has also been applied in the analysis of gene expression across diverse conditions [25]. For each chromatin peak, we calculated its relative accessibility in a given tissue type (i) as Ri = Ei/ΣE, where Ei represents the RPM (Reads Per Million) value for the peak in tissue i, and ΣE is the sum of RPM values across all tissues. The tissue specificity of chromatin accessibility was then quantified using the entropy score, calculated as H = − 1 * Σ (Ri * log2Ri), where H ranges from 0 to log2 (N), with N being the total number of tissues. An entropy score close to 0 indicates that a peak is highly specific to a particular tissue, while a score near log2(N) suggests that the peak is accessible across multiple tissues [26].

### Motif Analysis

Motif enrichment analysis was conducted to identify transcription factor binding site (TFBS) motifs within ATAC-seq peaks across tissues. Transcription factor motifs in tissue-specific peaks were identified using the findMotifsGenome.pl script from Hypergeometric Optimization of Motif EnRichment2 (HOMER2 version 5.0.1) with default settings to identify enriched sequence motifs [27]. To perform this analysis, we focus on known motifs from a vertebrate database. Background sequences were generated using the *Sus scrofa* reference genome (Sscrofa11.1) to account for genomic biases and ensure accurate motif enrichment detection. These background sequences provided a baseline for comparative analysis against the tissue-specific ATAC-seq peak sequences. Chromatin-accessible regions were analyzed to identify significantly enriched motifs, indicating the potential regulatory roles of the corresponding transcription factors. Enrichment was determined by comparing observed sequences within peaks to background sequences, with statistical thresholds applied to determine significance (*p* < 0.001).

### Differentially Accessible Regions Between Tissues

To detect differentially accessible regions (DARs), DiffBind (version 3.9.5) [28] was used, incorporating DESeq2 for differential peak analysis between tissues. DARs with a false discovery rate (FDR) < 0.05 were considered statistically significant. To visualize the distribution of DARs and their relationship to genomic features, sequencing tracks were examined using IGV (Integrative Genomics Viewer).

### Library Construction and RNA-Seq Data Pre-processing

RNA-Seq data from the same animals and tissues, obtained from our previous study (ArrayExpress accession E-MTAB-14452), were used. FastQC (version 0.11.7) was employed for quality assessment, evaluating per-base sequence quality, GC content and sequence duplication levels. Low-quality reads (mean Q-score < 30) and adapter-like sequences were removed using Trim Galore (version 0.6.7). High-quality paired-end reads were aligned to the Sus scrofa reference genome (Sscrofa11.1, ENSEMBL release 105) using HISAT2 (version 2.1.0) achieving a mapping efficiency of 98%. Read counting was performed using the HTSeq program (version 0.8.0). We calculated Pearson correlations between gene expression and chromatin accessibility at promoter regions and associations with *p* values < 0.05 were considered significant.

## Results

A total of 48 ATAC-seq libraries were generated from the pig’s limbic system and HPA/stress axis organs, including Amy, Hip, Tal, HT, PG and AG all derived from the same individuals. A standard pipeline, nf-core/atacseq within NEXTFLOW, was used for stringent quality filtering. The mean sequencing depth was 64.8 ± 11.3 million total reads, yielding 47.1 ± 8.4 million usable reads per sample, as detailed in Supp. Table 1. The average total number of peaks identified was 94,542.3 ± 17,625.7, with 11.6 ± 2.09% of these classified as TSS peaks (peaks located within − 2000 to + 2000 bp of transcription start sites, TSS) (Supp. Table1). The Fraction of Reads in Peaks (FRiP) score, a key ATAC-seq quality metric that evaluates the proportion of mapped reads falling within MACS2-identified peak regions, had an average value of 0.35 ± 0.06 across all samples indicating a good level of enrichment in peak regions (Supp. Table 1). Further peak annotation was performed using HOMER’s annotatePeaks.pl to classify peaks based on genomic features. On average, 11.7 ± 2.09% of the identified peaks were located within promoter regions (Supp. Table 1).

**Fig. 1:**
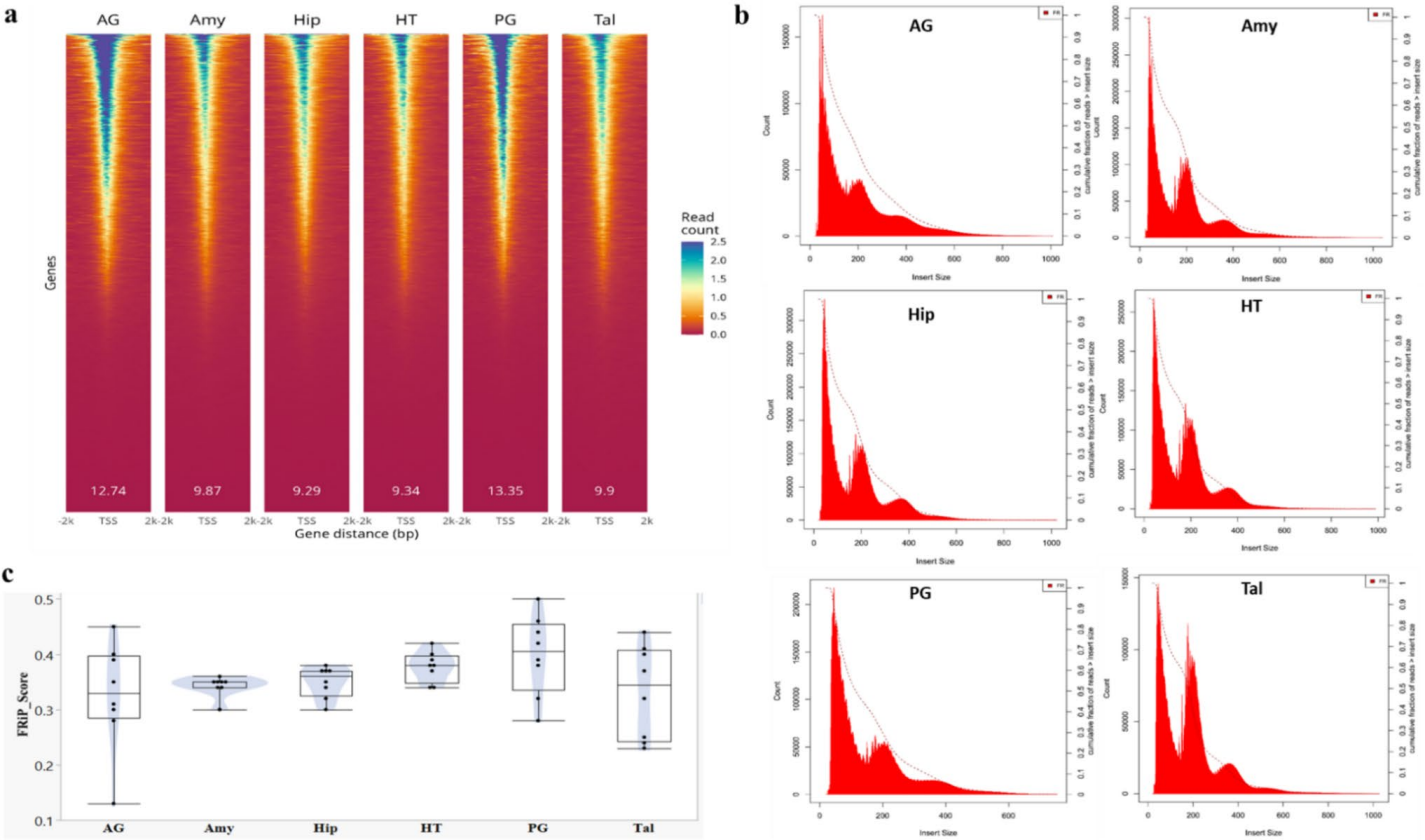
Tissue specificity of chromatin accessibility. **a** Heatmaps depicting ATAC-seq signals around all TSSs of a representative example of each tissue sorted by signal intensity. Signals shown for adrenal gland (AG), amygdala (Amy), hippocampus (Hip), hypothalamus (HT), pituitary gland (PG) and thalamus (Tal). White numbers represent mean read count enrichments at TSSs relative to surrounding ± 2 kb regions. **b** Fragment length distribution of ATAC-seq reads for a representative example of each tissue. **c** Fraction of Reads in Peaks (FRiP) distributions for all samples of each tissue

**Fig. 2:**
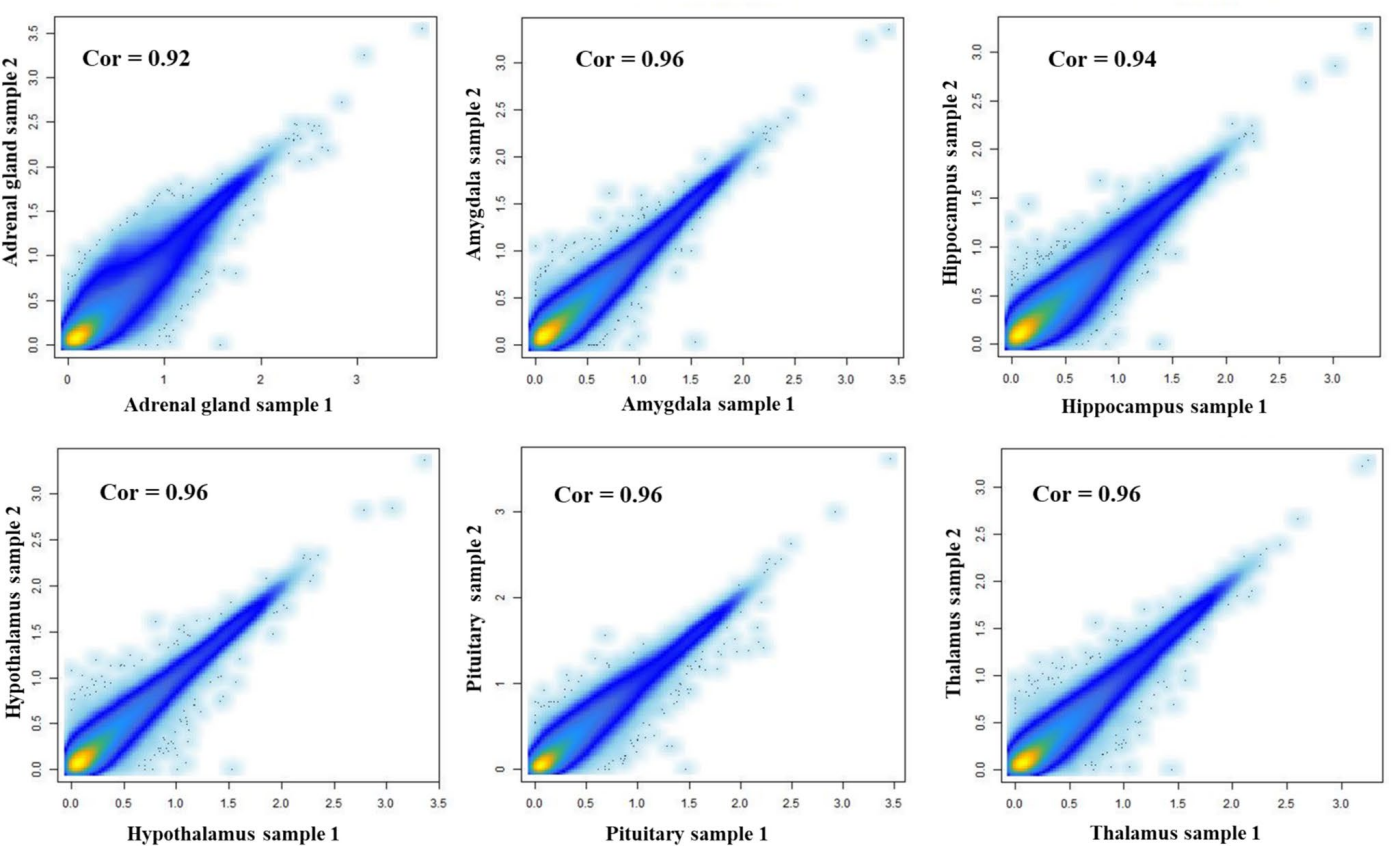
Scatter plots depicting the Pearson correlation between the first two ATAC-seq samples for each tissue. The X- and Y-axes represent log10 (RPM (Reads Per Million) values for samples 1 and 2, respectively

Summary statistics for each tissue across the eight individuals indicated that the proportion of usable reads ranged from 59% in AG to 83% in Hip (Table 1). AG tissue showed the highest variability in total reads, while it also exhibited the highest percentage of TSS peaks (14%) compared to other tissues (Table 1). ATAC-seq signal enrichment around the TSS (± 2 kb) for representative samples from each tissue is shown in Fig. 1a, along with the corresponding insert size distribution (Fig. 1b) for the same samples. The chromatin accessibility fragment distribution exhibits clear size periodicity corresponding to integer multiples of nucleosomes, indicating high library quality. The average of FRiP score in each tissue ranged from 0.33 in AG to 0.40 in PG (Fig. 1c) meeting the official standards established by Encyclopedia of DNA Elements (ENCODE) (https://www.encodeproject.org/atac-seq/). For evaluating reproducibility across datasets, a strong correlation between biological replicates in each tissue was observed (Fig. 2).

**Table 1:** ATAC-seq data and mapping statistics of 6 tissues including the amygdala (Amy), hippocampus (Hip), hypothalamus (HT), thalamus (Tal), adrenal gland (AG) and pituitary gland (PG)

Tissue	Total Reads (M)	Mapped reads (M)^a^	chrM reads (M)^b^	Usable reads (M)^c^	% Usable reads	Total peaks (K)^d^	% TSS peaks^e^
Amy	66.44 ± 8.7	64.99 ± 8.5	8.92 ± 4.3	49.61 ± 5.1	75.05 ± 5.8	106.00 ± 9.7	10.05 ± 0.7
Hip	66.03 ± 6.9	64.53 ± 6.7	2.93 ± 1.1	55.08 ± 5.6	83.46 ± 2.3	109.68 ± 16.3	10.00 ± 1.2
HT	64.96 ± 9.0	63.52 ± 8.9	4.51 ± 3.3	51.73 ± 6.6	79.90 ± 5.0	100.21 ± 9.5	11.10 ± 0.8
Tal	54.09 ± 9.9	52.71 ± 9.9	3.74 ± 3.4	43.78 ± 5.7	81.57 ± 4.6	84.72 ± 15.7	12.49 ± 1.9
AG	65.53 ± 17.7	63.46 ± 17.4	18.50 ± 11.2	37.66 ± 8.9	59.05 ± 10.1	75.07 ± 12.5	14.16 ± 2.3
PG	71.76 ± 6.7	70.17 ± 6.5	16.28 ± 7.6	44.65 ± 6.1	62.47 ± 8.2	91.17 ± 14.8	12.10 ± 1.8

^a^Mapped reads = total number of reads that map to the porcine genome

^b^chrM reads = number of reads that map to the mitochondrial genome

^c^Usable reads = number of mapped reads excluding low mapping quality, duplicate and mitochondria reads

^d^Total peaks = number of macs2 peaks

^e^% TSS peaks = percentage of peaks being within TSS region (-1000 bp <  = TSS <  = 100 bp) using Homer2; M for Million; K for Thousand and TSS for Transcription Start Site

### Tissue-Specific Chromatin Accessible Regions

**Fig. 3:**
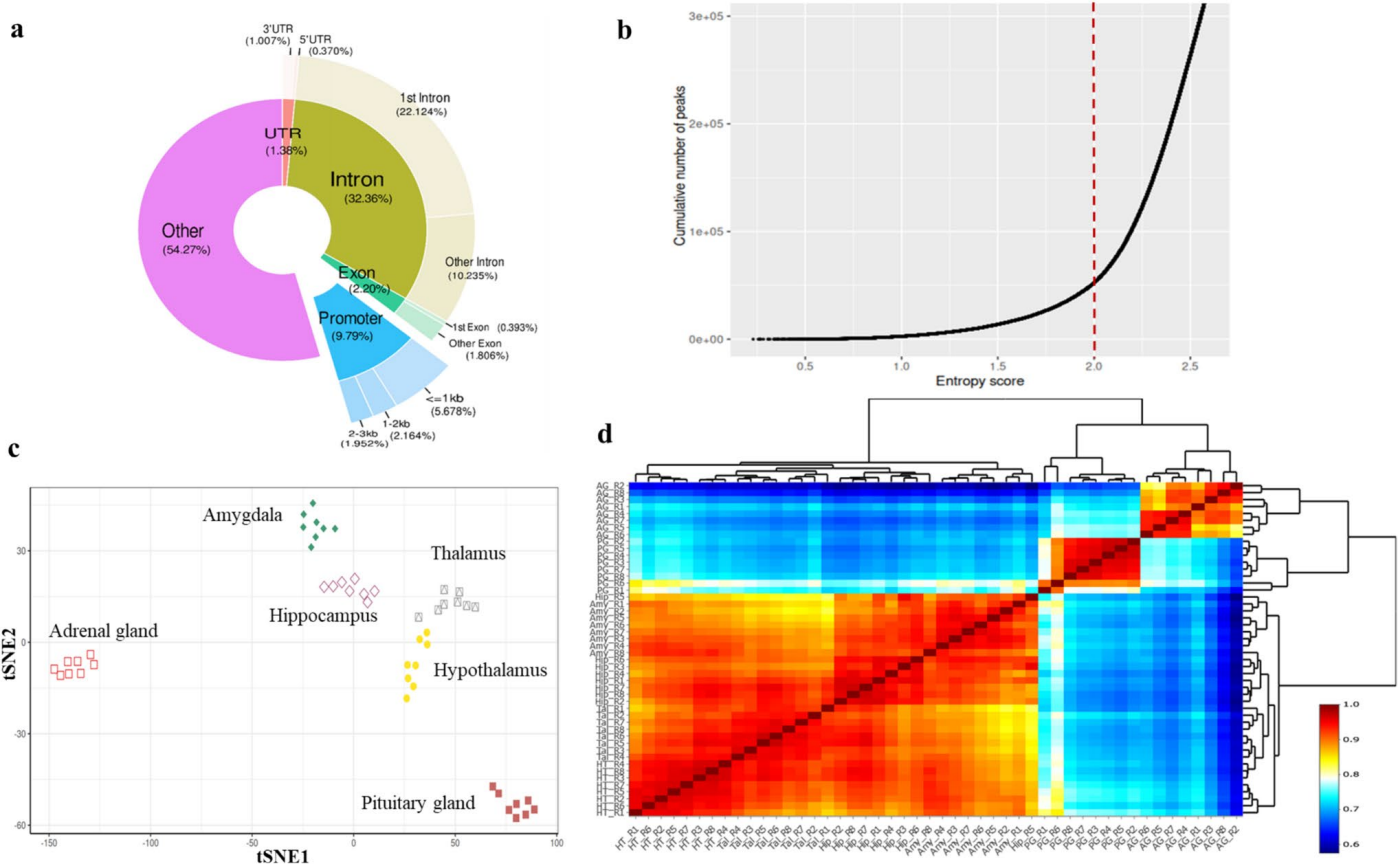
Comparisons of open accessibility regions in various brain and endocrine tissues. **a** Distribution of open accessibility regions across genomic contexts, categorized as promoter (3 kb upstream of TSS), UTR (5′ UTR and 3′ UTR), exon (first exon and other exons), intron (first intron and other introns), and other. **b** Distribution curve of peak entropy scores. **c** t-SNE projection of ATAC-seq profiles on tissue-specific peaks. **d** Heatmap clustering of correlations across all 48 ATAC-seq profiles

In our analysis, we identified a total of 321,584 consensus peaks representing open chromatin regions across various samples and tissues in the pig genome. Approximately 9.8% (31,496/321,584) of these peaks were located on promoter regions of coding or noncoding transcripts (Fig. 3a). About 31% (99,576/321,584) of these peak were located on/near noncoding transcripts. Next, we determined tissue-specific chromatin-accessible regions using Shannon entropy to compute a tissue specificity index for each peak (see “Methods” section). Peaks were classified as tissue-specific if they met two criteria: (1) an entropy score (H) of less than 2 (51,130 peaks) (Fig. 3b), indicating low dispersion across tissues, and (2) a relative accessibility (Ri) of at least 0.33 (51,056 peaks), meaning that at least 33% of the peak signal was concentrated in a single tissue. Applying these criteria, 6641, 4257, 112, 27,434, 15,483 and 1157 tissue-specific chromatin accessible regions were identified in the Amy, Hip, HT, AG, PG and Tal, respectively (Supp. Table 2). Approximately 3397 peaks are located in promoter regions (± 3 kb), with the majority (52%) associated with protein-coding genes and 43% with long non-coding RNA (lncRNA).

A t-SNE (t-distributed Stochastic Neighbor Embedding)–based clustering plot, which applies a machine learning algorithm to reduce high-dimensional data into two or three dimensions for visualization, was used to illustrate tissue-specific chromatin-accessible regions across all 48 samples, as shown in Fig. 3c. A higher correlation score was observed between samples within the same tissues. The heatmap (Fig. 3d) revealed a clear separation between endocrine glands (PG and AG) and brain regions, with HT and Tal clustering together, as did Hip and Amy. The observed separation indicates distinct chromatin-accessibility profiles, reflecting differences in their transcriptional activity and regulatory elements tailored to their specific hormonal roles.

### Motif Enrichment in Tissue-Specific Chromatin Accessible Regions and Expression of Corresponding Transcription Factors

**Fig. 4:**
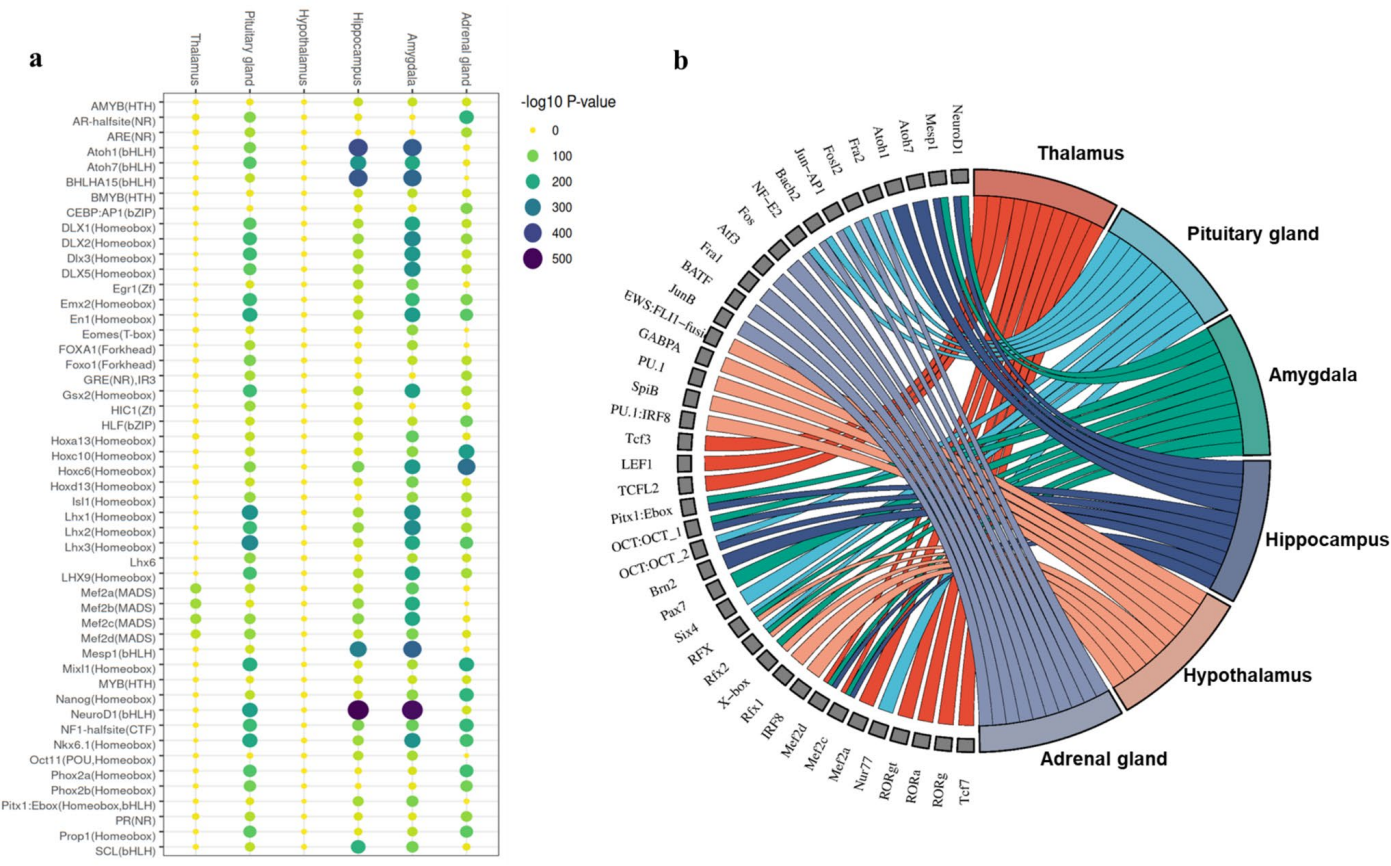
Transcription factor binding site motifs in tissue-specific chromatin accessible regions. **a** Enrichment of 50 transcription factor motifs with the highest coefficient of variation (CV) and mean enrichment values greater than 20 across tissues. Color and size of points indicate motif enrichment *p*-values. **b** Top 10 significantly enriched (*p* < 0.001) transcription factor binding site motifs with the highest fold enrichment for each brain and endocrine tissue

**Fig. 5:**
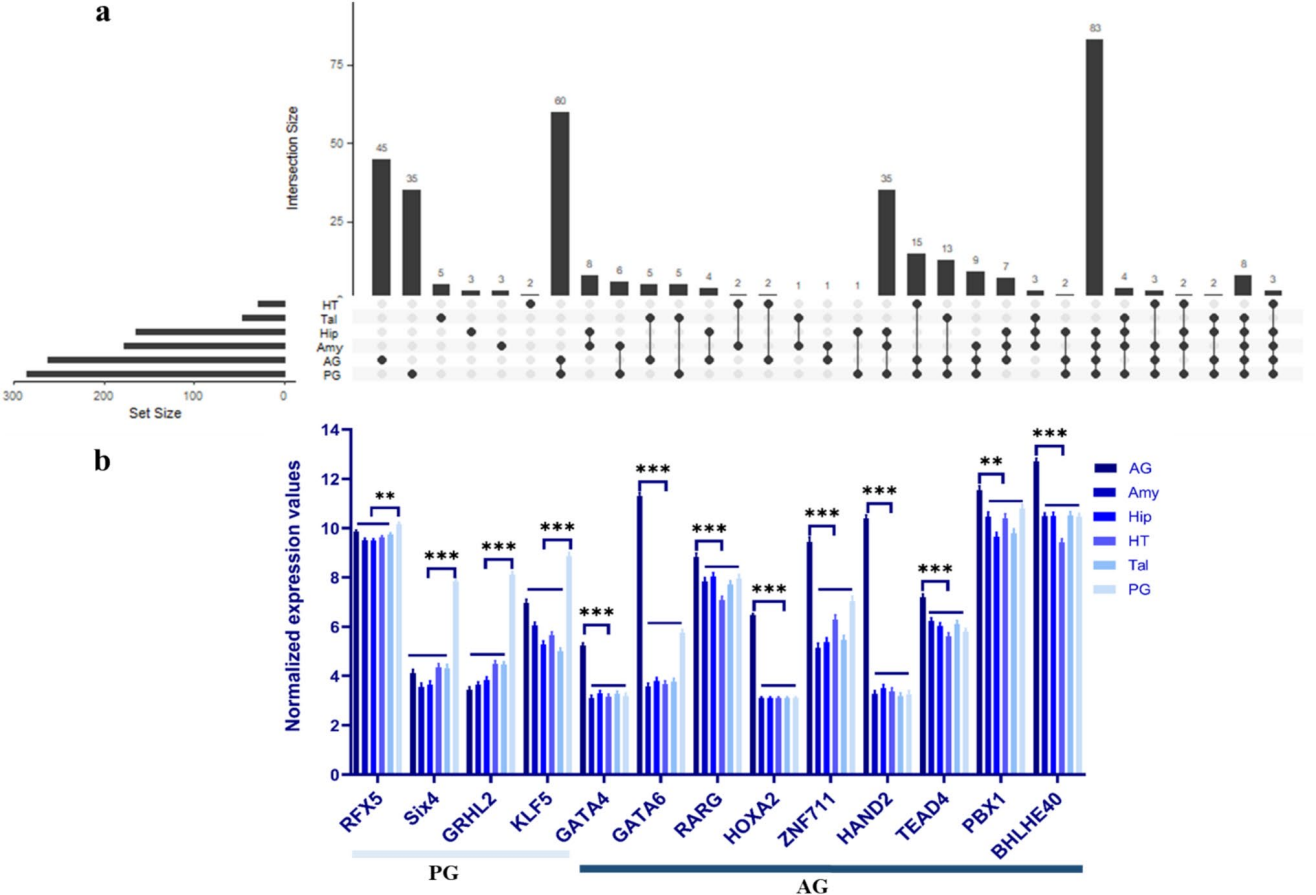
Predicted motifs from tissue-specific chromatin accessible regions. **a** UpSet plot showing the number and overlap of significantly enriched motifs (*p* < 0.001) identified in each tissue: adrenal gland (AG), amygdala (Amy), hippocampus (Hip), hypothalamus (HT), pituitary gland (PG) and thalamus (Tal). **b** Expression patterns of transcription factors corresponding to predicted tissue-specific motifs in the pituitary gland (*PG*; *RFX5*, *SIX4*, *GRHL2* and *KLF5*) and adrenal gland (AG; *GATA4*, *GATA6*, *RARG*, *HOXA2*, *ZNF711*, *HAND2*, *TEAD4*, *PBX1* and *BHLHE40*). **, *** indicate statistical significance at *p* < 0.05 and *p* < 0.001

Motif enrichment analysis revealed transcription factor binding sites that are potentially involved in the regulation of gene expression in a tissue-specific manner, based on changes in chromatin accessibility. We generated a motif enrichment matrix, where each row represents the *p* value of a motif and each column represents a tissue. In total, 377 motifs were found to be significant enriched at least in one tissues (*p* ≤ 0.001) (Supp. Table 3). The top 50 motifs with the highest coefficient of variation (CV) values and mean enrichment scores greater than 20 are shown in Fig. 4a. The top 10 transcription factor binding motifs with the highest fold enrichment in each tissue are shown in Fig. 4b. The overlap of significant motifs across different tissues is shown in Fig. 5a using an UpSet plot. A total of 93 motifs were identified as enriched in only one tissue-specific chromatin accessible region, including 45 motifs in AG, 35 in PG, 5 in Tal, 3 in Hip, 3 in Amy and 2 in HT. To assess the biological relevance of the predicted motifs, we analyzed bulk RNA-seq data from the same samples to determine whether the corresponding transcription factors are expressed in the respective tissues. Among the 93 predicted tissue-specific motifs, we found gene expression support for 43 of the corresponding transcription factors. However, some of these TFs are expressed in more than one tissue. To address this, we further filtered for TFs with significantly higher expression in the corresponding tissue compared to all others. This analysis identified 13 transcription factors with both motif enrichment in tissue-specific chromatin regions and tissue-specific gene expression, 9 (*GATA4*, *GATA6*, *RARG*, *HOXA2*,* ZNF711*, *HAND2*, *TEAD4*,* PBX1* and *BHLHE40*) in AG and 4 (*RFX5*,* SIX4*, *GRHL2* and *KLF5*) in PG (Fig. 5b).

**Fig. 6:**
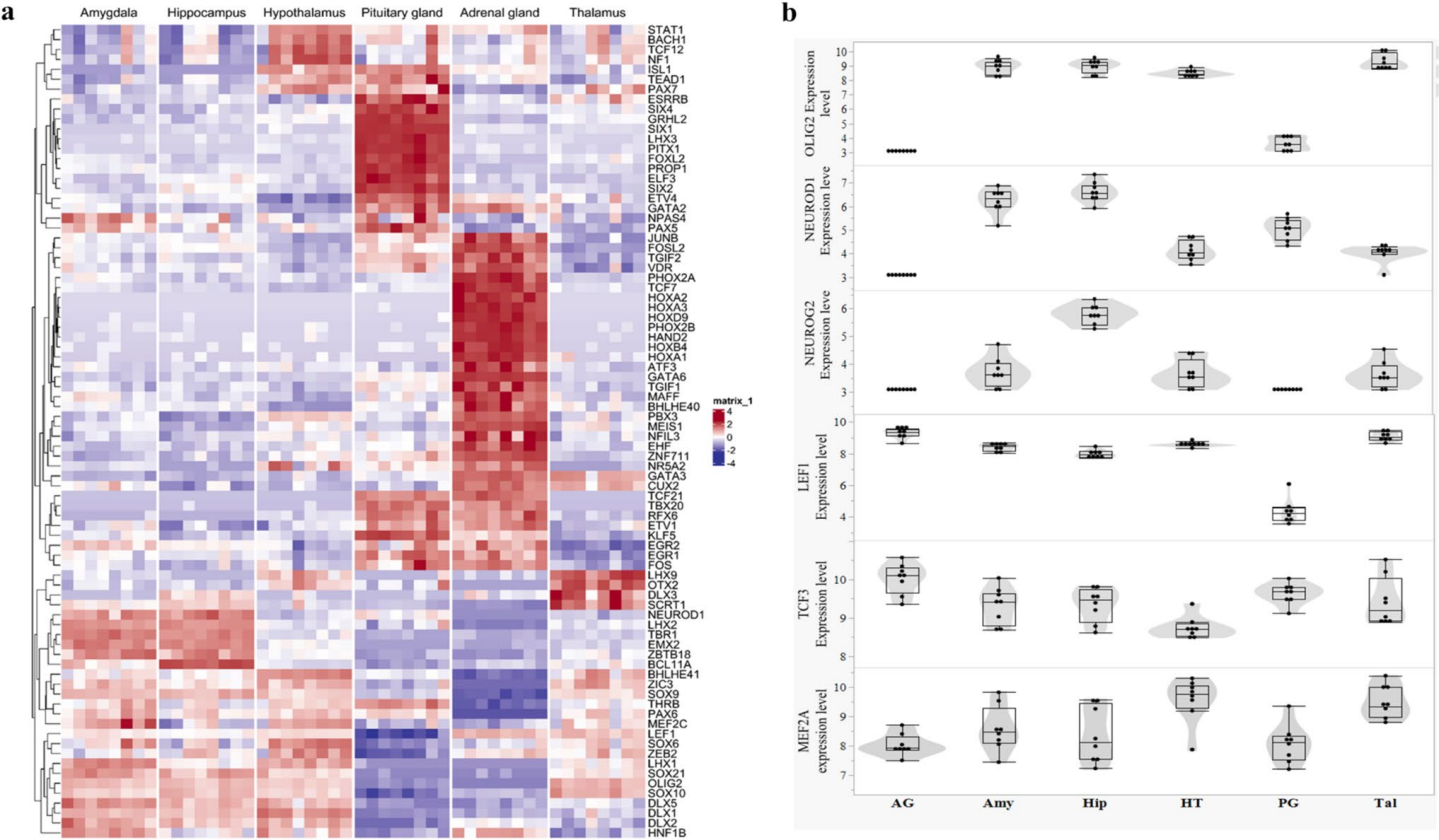
Transcription factor expression across brain and endocrine tissues **a** heatmap clustering of transcription factor expression across tissues. The colored bars represent genes with variable expression levels among the different tissues. **b** Expression patterns of selected transcription factors—*OLIG2*, *NEUROD1*, *NEUROG2*, *LEF1*, *TCF3* and *MEF2 A*—in brain regions (amygdala [Amy], hippocampus [Hip], hypothalamus [HT], and thalamus [Tal]) and endocrine tissues (adrenal gland [AG] and pituitary gland [PG])

In general, of the 377 predicted motifs, 221 (approximately 58%) correspond to transcription factors that were expressed in at least one tissue. The expression patterns of these transcription factors are shown in Fig. 6a. The most highly enriched motifs in the thalamus include those for lymphoid enhancer-binding factor 1 (LEF1), transcription factor 3 (TCF3), MEF2 family (MEF2 A, MEF2B, MEF2 C and MEF2D), Nuclear receptors (NR) (RORa, RORg and RORgt) and SOX Family (SOX1, SOX3 and SOX10). Relative higher transcript abundance of LEF1, TCF3 and MEF2 A in thalamus compared to the other tissues was found on Fig. 6b. Highly significantly enriched motifs were similarly found between Amy and Hip and include oligodendrocyte transcription factor 2 (Olig2), neuronal differentiation 1 (NeuroD1) and Neurogenin 2 (NeuroG2). The expression level of OLIG2, NEUROD1 and NEUROG2 was also higher in Hip (Fig. 6b) which confirm and validated our predicted motifs. We also found TCF4 and Mesp1 enrichment motifs particular in Hip. The most enriched motifs in the AG include Fos, Atf3, Fra1, BATF, AP-1, JunB, Fra2 and Fosl2. The PG is essential for regulating key physiological processes, acting as a bridge between the central nervous system and the endocrine system to coordinate various bodily functions. Notably, most of the motifs enriched in the PG were also found to be significant in AG. We observed an enrichment of the RFX family (Rfx1, Rfx2, RFX) and NKX family (Nkx2.1, Nkx2.2) transcription factors in the HT.

### Integration of ATAC-seq and RNA-seq

**Fig. 7:**
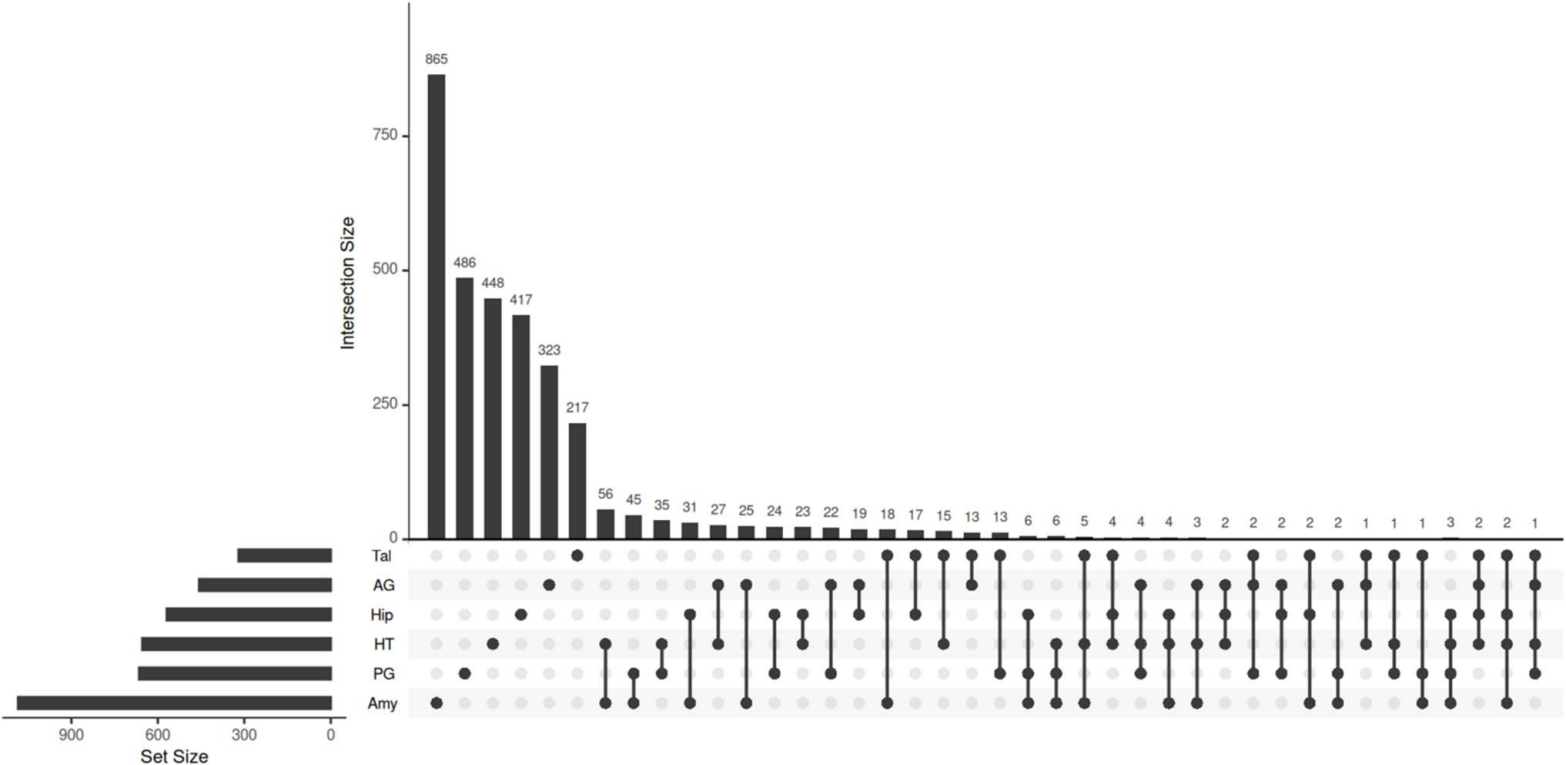
Correlation between accessibility regions and expression patterns in each tissue. An UpSet plot showing the number of genes with a positive correlation between promoter accessibility regions and the expression levels of the same genes in the adrenal gland (AG), amygdala (Amy), hippocampus (Hip), hypothalamus (HT), pituitary gland (PG) and thalamus (Tal)

We integrated our previously generated bulk RNA-seq data from the same samples, allowing us to directly assess whether identified chromatin alterations led to transcriptional changes in the transcription factors (TFs) and their target genes. We calculated Pearson correlations between gene expression and chromatin accessibility at promoter regions. We defined an ATAC-seq peak within ± 3 kb of a gene’s TSS as a promoter (proximal regulatory element), encompassing 31,469 peaks corresponding to 20,467 transcripts. Of these, approximately 15,560 transcripts from RNA-seq data corresponded with ATAC-seq-derived transcripts. We focused on positive correlations between RNA-seq data and accessible regions, using only the result with the highest correlation coefficient for each gene. When peaks were located in promoter regions of the same transcripts, these positive correlations were classified as *cis* correlations. In total, we observed 3205 pairs of peaks and corresponding transcript levels which showed at least one positive cis correlation with *p* values < 0.05 and R = 0.70 in each tissue. Most correlation of promoter accessibility and their transcripts was found in Amy (1085) followed by PG (665), HT (654), Hip (569), AG (457) and Tal (320). The intersection plot showed a number of significant correlations between accessible peaks and transcription rates in each of the tissues (Fig. 7).

### Differentially Accessible Regions Between Brain Regions and Endocrine Tissues Along the Hypothalamic–Pituitary–Adrenal Axis

**Fig. 8:**
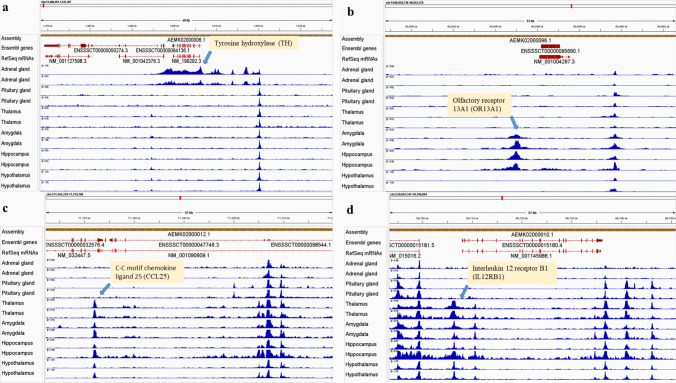
ATAC-seq signals in selected pig tissues with tissue-specific activity. **a** The height of the peaks represents the density distribution of ATAC-seq reads around the promoter of Tyrosine Hydroxylase (*TH*). The promoter region of this gene, which is the rate-limiting step in tyrosine metabolism, exhibits open chromatin accessibility in the adrenal gland compared to other brain tissues. **b** Open chromatin accessibility in the promoter of Olfactory Receptor 13 A1 (*OR13 A1*) in the amygdala and hippocampus. **c** Open chromatin accessibility in the promoter of C–C motif chemokine ligand 25 (*CCL25*), and **d** interleukin12 receptor subunit beta 1 (*IL12RB1*). Both of these transcripts belong to the cytokine-cytokine receptor interaction pathway, which is more enriched and open chromatin accessibility in brain regions compared to endocrine tissue (adrenal gland and pituitary gland)

Pairwise comparisons of ATAC-seq data were conducted using DiffBind to identify differential peaks between tissues. A total of 311,642 peaks were identified as DARs in at least one tissue comparison, applying an FDR threshold of < 0.05 (Table 2). The number of DARs between brain regions is summarized in Table 2. Comparisons involving brain tissues, including Amy, Hip, HT and Tal, revealed greater differences relative to AG and PG, with a higher number of DARs. The number of DARs ranged from 177,815 (HT vs. PG) to 215,635 (Amy vs. AG), while comparisons between AG and PG identified 160,304 peaks. In contrast, similar numbers of DARs were observed between brain regions such as Amy, Hip, HT and Tal, with DARs ranging from 37,785 (Amy vs. Hip) to 109,847 (Amy vs. Tal). Further analysis focusing on peaks located within promoter regions (± 3 kb from the TSS) with |FC|> 2 and FDR < 0.05 revealed 14,089 peaks in at least one tissue comparison (Supp. Table 4). The genes associated with DARs located within promoter regions were submitted for pathway enrichment analysis (Supp. Table 5 and Table 2). We observed open accessibility in promoter regions of genes enriched in Tyrosine metabolism (*FAH*, *DBH*, *ADH1 C*, *HPD*,* PNIMT*, *MAOB*, *AOX1*, *LOC100520329* and *TH*) and Cortisol synthesis and secretion (*ADCY4*, *ADCY7*, *CACNA1I*,* CREB3L3*, *CYP11 A1*,* CYP11B2*, *CYP17 A1*, *CYP21 A1*, *HSD3B1*, *MRAP*, *NR5 A1* and *STAR*) in AG when compared to other tissues. Open accessibility was found in the promoter region (≤ 1 kb) of *TH* in AG tissue (Fig. 8a). Fewer DARs were identified in promoter regions when comparing Hip with Amy, HT and Tal, or HT with Tal. No pathway enrichment analysis reached an FDR < 0.05 in these comparisons. However, an interesting enrichment of the olfactory transduction pathway was observed when comparing DARs in the promoter regions of Amy with HT or Tal. Open accessibility region of olfactory receptor family 13 subfamily A member 1 (*OR13 A1*) located on promoter region (1–2 kb) was found in Amy and Hip (Fig. 8b). Differentially accessible regions in other brain regions, including the Amy, Hip, HT and Tal, show enrichment in cytokine-cytokine receptor interaction and neuroactive ligand-receptor interaction pathways when compared to the PG or AG. Examples of open chromatin accessibility in the promoter regions of transcripts involved in the cytokine-cytokine receptor interaction pathway include C–C motif chemokine ligand 25 (*CCL25*) and interleukin-12 receptor subunit beta 1 (*IL12RB1*) (Fig. 8c and d). Both of these transcripts belong to the cytokine-cytokine receptor interaction pathway, which exhibits greater enrichment and open chromatin accessibility in brain regions compared to endocrine tissues (AG and PG). In addition to these important pathways when comparing these brain regions, other pathways, including complement and coagulation cascades, were also prominent when compared with the AG.

**Table 2:** The number of differentially accessible regions (DARs) and top 3 enriched pathways between brain regions with FDR < 0.05, DARs located within promoter regions (± 3 Kb from the TSS) and a |fold change|> 2

Comparison	Number of DARs	DARs in promoter region	Pathway enrichment
Amy vs. Hip	37,785	2	
Amy vs. HT	80,257	560	Olfactory transduction
Amy vs. PG	207,263	6185	Cytokine-cytokine receptor interaction, Neuroactive ligand-receptor interaction, Viral protein interaction with cytokine and cytokine receptor
Amy vs. AG	215,635	7259	Neuroactive ligand-receptor interaction, Complement and coagulation cascades, Cytokine-cytokine receptor interaction
Amy vs. Tal	109,847	981	Olfactory transduction
Hip vs. HT	74,381	459	
Hip vs. PG	211,911	6316	Cytokine-cytokine receptor interaction, Neuroactive ligand-receptor interaction, Rap1 signaling pathway
Hip vs. AG	215,098	7303	Cytokine-cytokine receptor interaction, Neuroactive ligand-receptor interaction, Complement and coagulation cascades
Hip vs. Tal	63,703	754	
HT vs. PG	177,815	5395	Cytokine-cytokine receptor interaction, Neuroactive ligand-receptor interaction, Viral protein interaction with cytokine and cytokine receptor
HT vs. AG	192,783	7303	Cytokine-cytokine receptor interaction, Neuroactive ligand-receptor interaction, Complement and coagulation cascades
HT vs. Tal	46,962	50	
AG vs. PG	160,304	4921	Neuroactive ligand-receptor interaction, Calcium signaling pathway, Ovarian steroidogenesis
Tal vs. PG	183,757	5529	Cytokine-cytokine receptor interaction, Neuroactive ligand-receptor interaction, Calcium signaling pathway
AG vs. Tal	186,831	6330	Complement and coagulation cascades, Neuroactive ligand-receptor interaction, Osteoclast differentiation

Abbreviations: *Amy *amygdala, *Hip *hippocampus, *Tal *thalamus, *HT *hypothalamus, *PG *pituitary gland, *AG *adrenal gland

## Discussion

Pig brains share key anatomical similarities with human brains, and their large body size enables the use of clinically available imaging equipment and surgical techniques to study various neurological conditions or neurodevelopment [29–31]. Structurally, pigs possess gyrencephalic brains that resemble the human brain in cortical organization, gray-to-white matter ratios and the expression of biomarkers such as GFAP/clusterin, which are relevant to neurodegenerative diseases [32, 33]. Furthermore, in specific brain regions—including the cerebellum and hypothalamus—the pig’s global gene expression profile more closely resembles that of humans than that of mice, supporting the pig as a potentially superior model for investigating a broad range of brain functions [34]. The pig genome, as reported by the Wellcome Trust Sanger Institute, spans approximately 2.7 Gb (gigabases) (https://www.sanger.ac.uk/data/pig-genome/). Assuming an average peak size of 300 base pairs, these open accessibility regions detected in this study collectively cover about 3.5% of the entire pig genome. This finding aligns well with observations in human genomics, where accessible regions of DNA typically represent around 3% of the genome [35]. Despite their relatively small footprint, these regions often play crucial roles in regulating gene expression. The similarity in the proportion of accessible chromatin between pig and human genomes underscores the conservation of genomic regulatory architecture across mammalian species.

Endocrine glands (PG and AG) and brain regions revealed a clear separation in heatmap, while HT and Tal clustering together, as did Hip and Amy. The observed clustering pattern highlights the functional specialization of these tissues. HT and Tal, both subcortical regions, share functional and anatomical proximity, which may explain their similar chromatin-accessible landscapes, potentially linked to their roles in homeostasis regulation, sensory signal relay and neural integration. A small number of tissue-specific chromatin-accessible regions (112) in HT was observed. Their similar regulatory landscapes and shared roles in central neural processing may result in a higher degree of overlap in accessible chromatin, reducing the number of regions uniquely accessible in the hypothalamus. Similarly, Hip and Amy, both part of the limbic system, exhibited comparable chromatin profiles, likely reflecting their shared involvement in memory consolidation (Hip) and emotional processing (Amy), two processes that frequently interact within brain networks. This overlap is supported by their close clustering in the chromatin accessibility heatmap, suggesting coordinated epigenomic regulation rather than highly divergent, tissue-specific signatures.

However, some of the predicted tissue-specific motifs and corresponding expression patterns observed in this study align with previously reported biological roles. For example, *GRHL2*, identified as a specific motif and expressed in the pituitary gland, has also been reported as a novel progenitor cell marker in the developing pituitary, contributing to progenitor cell function and maintenance [36]. Similarly, *KLF5* has been described as a key mediator of the stress response [37, 38]. In the adrenal gland, predicted tissue-specific motifs and expression patterns for *GATA4, GATA6, HAND2*, and *PBX1* are consistent with their known roles as key regulators of adrenal steroidogenesis, structural development and metabolic function [39–42]. Recent studies have confirmed and expanded upon the established role of HAND2 in the differentiation and maintenance of chromaffin cells, as well as in the regulation of catecholamine biosynthetic enzymes such as tyrosine hydroxylase (TH) and dopamine β-hydroxylase (DBH) [42, 43]. Notably, in this study, we observed open chromatin accessibility in the promoter regions of genes involved in tyrosine metabolism, including *TH* and *DBH*. As shown in Fig. 8a, the promoter region of *TH*—which encodes the rate-limiting enzyme in tyrosine metabolism—exhibited open chromatin accessibility in the adrenal gland, but not in other brain tissues.

Overlap of enriched motifs in tissue-specific accessible regions included several whose corresponding transcription factors were also overlap expressed in the same tissue. For example, one of the top enriched motifs in thalamus-accessible regions was LEF1, whose corresponding transcription factor showed its highest expression in the thalamus. Interestingly, the consensus motif of LEF1 is also enriched in open chromatin regions of the AG, and LEF1 expression is second highest in AG, followed by lower levels in Amy, Hip and HT, with the lowest expression observed in PG (Fig. 5b). This constellation of motif enrichment and corresponding transcription factor expression reflects the complex relationship between chromatin accessibility, transcription factor abundance and gene regulation in bulk tissue analyses. In the case of LEF1 across the tissues studied, chromatin accessibility may have a greater impact on tissue-specific expression profiles than the mere presence of the transcription factor. Single-cell approaches may help to further disentangle the respective contributions of chromatin accessibility and transcription factor expression to the regulation of specific genes and pathways. After birth, *Lef1* mRNA levels decline sharply in the brain, remaining detectable only in the thalamus [44, 45]. Together with beta-catenin, LEF1/TCF transcription factors enhance neuronal excitability, not only through localized actions at the synapse but also by activating gene expression in thalamic neurons [46, 47].

The myocyte enhancer factor 2 (MEF2) family of transcription factors consists of four highly conserved members that play a crucial role in the nervous system [48]. In vertebrates, the MEF2 isoforms are encoded by distinct genes: *MEF2 A*, *MEF2B*, *MEF2 C* and *MEF2D* [49]. We observed MEF2 Family motifs in PG, Amy and Hip with varying levels of significance. Among these, *MEF2 A* is predominantly expressed in the developing thalamus compared to other isoforms, highlighting its critical role in thalamic functional development [48]. Specific motifs in Amy and Hip include oligodendrocyte transcription factor 2 (*Olig2*), which is crucial for the specification and differentiation of oligodendrocytes, astrocytes and neurons during development [50, 51]. Neuronal differentiation 1 (NeuroD1) and Neurogenin 2 (NeuroG2) are involved in neuronal differentiation and promote neurogenesis [52]. Recent studies reveal that chick Neurog2 and NeuroD1 are critical to the successful development of the trigeminal ganglion and its nerve branches [52]. Additionally, the signatures of several transcription factors, such as Olig2, NeuroD1, TCF4, and NeuroG2, were found to be enriched in Alzheimer’s disease-associated accessible chromatin regions within hippocampal tissues [53]. Motifs significantly enriched in Amy belong to *DLX2* and *DLX5* (Distal-Less Homeobox 2 and 5), which are involved in forebrain development, particularly in the differentiation of GABAergic interneurons [54]. Lhx1 and Lhx2 are LIM homeobox transcription factors and are involved in neuronal differentiation and axonal guidance [55]. Atonal BHLH transcription factor 1 (Atoh1) is required for many sensory neuronal lineages in the hindbrain and plays a functional role in migration [56].

Both the pituitary gland and adrenal gland, central components of the HPA axis, showed enrichment for motifs associated with the activator protein-1 (AP-1) complex—including Fos, Fra1, Fra2, JunB and Jun-AP1. These transcription factors, part of the bZIP family, are well-established regulators of gene expression in response to stress, immune signaling and growth factors [57]. AP-1 DNA-binding activity is modulated in a tissue- and development-specific manner, with high basal levels during early brain development and elevated activity in the adrenal glands of adult rats [58]. Additionally, previous studies comparing pituitary glands from different pig breeds (e.g. Bama Xiang vs. Large White) have reported similar enrichment of AP-1-related motifs—including JunB, ATF3, FRA1 and BATF—further supporting the functional relevance of these transcription factors in the regulation of the HPA axis [59]. Pit1 (pituitary-specific positive transcription factor 1 also known as POU1 F1) is a well-studied transcription factor critical for pituitary development and function [60]. PG-enriched motifs also include *ZEB1*, which has been linked to anovulation and infertility in mice lacking the microRNAs that target ZEB1 in the pituitary gland [61]. In our study, we identified accessible regions for *ZEB1* motifs, suggesting another layer of epigenetic regulation. Notably, the enrichment of binding motifs for the RFX, NKX and MEF families occurred in the mediobasal hypothalamus, where chromatin accessibility was shown to be altered by photoperiod regulation [62].

Nkx transcription factors play critical roles in neuronal differentiation and hypothalamic development. In the spinal cord, the combinatorial expression of Nkx genes, including Nkx2.2, guides ventral neuronal identity [63]. Of particular relevance to the HPA axis, NKX2.1 is essential for early hypothalamic morphogenesis and is especially involved in the development of melanocortin neurons and regulation of POMC expression, a gene central to energy balance and body weight control [64]. We observed enrichment of NKX2.1, NKX2.2 and NKX2.5 motifs along HPA axis, with NKX2.5 primarily recognized for its role in cardiac development [65, 66]. Additionally, we identified ETV1, ETV2 and ETV4 motifs enriched in chromatin-accessible regions of HPA axis tissues. These ETS family transcription factors regulate POMC transcription in pituitary corticotrophs and melanotrophs [67], and ETV4 has been linked to antidepressant response [68], further supporting their relevance in stress regulation. We also observed enrichment of ELF1, ELF3 and ELF4—other ETS family members—known for their roles in immune signaling, cytokine response and cellular regulation [69–71], suggesting potential involvement in immune–endocrine cross-talk within the HPA axis. Together, the results are supported by numerous scientific references demonstrating the biological importance of these motifs for neuronal development, differentiation, and functional regulation. Our study not only provides maps of the transcriptional landscapes in different brain regions in pig, but also offers important insights into how specific transcription factors contribute to tissue-specific gene expression and corresponding neurological functions.

A small number of correlated features between gene expression and chromatin accessibility changes was observed. Generally, changes in chromatin accessibility tend to align with changes in transcription, as significant alterations in transcriptional output often correspond to widespread changes in the accessibility of multiple loci within the surrounding chromatin [72]. However, some studies indicate that many changes in gene expression can occur independently of local chromatin accessibility changes, particularly in the context of single-factor perturbations [73]. Furthermore, another study observed that only a small fraction of chromatin accessibility changes are linked to expression changes in nearby genes [74]. Together, gene expression is influenced by a multitude of factors beyond chromatin accessibility, including transcription factors, post-transcriptional modifications and environmental conditions. This complexity contributes to the limited direct correlation between chromatin accessibility and gene expression.

Furthermore, DARs between brain or endocrine tissues and their putative functional impacts were investigated. We observed open chromatin accessibility in the promoter regions of genes involved in tyrosine metabolism and cortisol synthesis in the AG compared to other tissues. Notably, the promoter region of tyrosine hydroxylase (*TH*), which is key in catecholamine synthesis, showed open accessibility in AG. The protein encoded by TH is involved in the conversion of tyrosine to dopamine which is the rate-limiting enzyme in the synthesis of catecholamines, hence playing a key role in the physiology of adrenergic neurons [75]. An enrichment in the olfactory transduction pathway was found in the Amy compared to the HT or Tal. The Amy and Hip are interconnected with the primary olfactory cortex, influencing behaviors related to olfactory perception [76]. While the HT and Tal are important brain regions with various functions, the amygdala’s direct involvement in olfactory processing [77], emotional evaluation of odours [78] and mediation of innate olfactory responses [79] makes the enrichment of olfactory transduction genes in this region biologically meaningful and consistent with its functional role in the olfactory system. Neuroactive ligand-receptor interactions are crucial for neurological functions and are associated with various neurological diseases [80, 81]. Cytokine-cytokine receptor interactions play a vital role in brain-body communication [82]. Recent evidence suggests that cytokines signal the brain to produce neurochemical, neuroendocrine, neuroimmune, and behavioral changes [83]. Complement and coagulation proteins are involved in signaling between the immune system and the brain disorder [84]. This communication is more relevant in brain regions than in the adrenal gland.

## Conclusion

This study provides novel insights into the epigenetic landscape of key brain regions and adrenal tissues in pigs by mapping chromatin accessibility across components of the hypothalamic–pituitary–adrenal (HPA) axis and limbic system. Using ATAC-seq, we characterized tissue-specific chromatin profiles and identified enriched transcription factor binding motifs within accessible regulatory regions. These findings reveal distinct epigenetic signatures in higher-order brain structures involved in neuroendocrine regulation, as well as in endocrine tissues responsible for stress hormone production. Our analysis uncovered differentially accessible regions and associated gene pathways that are specific to either brain or adrenal tissues. These chromatin accessibility profiles, generated from healthy, untreated animals, provide a foundational reference for future research into regulatory mechanisms underlying neuroendocrine function in pigs. This resource may inform future studies aimed at genetic improvement targeting animal welfare and behavior in agricultural settings, given the functional relevance of these regions to stress response and subjective states based on cognitive and emotional experiences and evaluations. While this study provides valuable insight into the chromatin accessibility landscape of brain and endocrine tissues, several limitations should be considered when interpreting the results, particularly regarding sex, age and breed. The analysis was limited to female pigs due to logistical constraints and standard meat production practices, in which male pigs are routinely castrated to prevent boar taint. However, given known sex differences in neuroendocrine regulation, including both sexes in future studies will enhance generalizability. The use of bulk tissue samples limits cell type–specific resolution, which is particularly relevant in complex tissues like the brain. Moreover, the study was conducted in healthy animals under standard conditions, reflecting a normal physiological, emotional and behavioural state and providing baseline data in the absence of disease or challenge models. Future work incorporating single-cell approaches and behavioural challenge models involving stressful or emotionally arousing conditions will be essential to fully understand neuroendocrine regulation and its relevance to animal welfare and translational research.

## Supplementary Information

Below is the link to the electronic supplementary material. 

ESM1 (XLSX 12.3 MB)

## Data Availability

Publicly available datasets were analyzed in this study. The data can be found here: E-MTAB-14452 for RNA seq data and E-MTAB-14923 (https://www.ebi.ac.uk/biostudies/arrayexpress/studies/E-MTAB-14923) for ATAC-seq data. Other data used to support the findings of this study are included within the article and the supplementary information files.
